# Pentoxifylline Attenuates Arsenic Trioxide-Induced Cardiac Oxidative Damage in Mice

**DOI:** 10.1155/2021/6406318

**Published:** 2021-01-07

**Authors:** Atefeh Gholami, Sara Ataei, Davoud Ahmadimoghaddam, Navid Omidifar, Amir Nili-Ahmadabadi

**Affiliations:** ^1^Medicinal Plants and Natural Products Research Center, Hamadan University of Medical Sciences, Hamadan, Iran; ^2^Department of Pharmacology and Toxicology, School of Pharmacy, Hamadan University of Medical Sciences, P.O. Box 8678-3-65178, Hamadan, Iran; ^3^Department of Clinical Pharmacy, School of Pharmacy, Hamadan University of Medical Sciences, Hamadan, Iran; ^4^Clinical Education Research Center, Department of Pathology, School of Medicine, and Biotechnology Research Center, Shiraz University of Medical Sciences, Shiraz, Iran

## Abstract

This study was undertaken to evaluate the therapeutic potential effect of pentoxifylline (PTX) against arsenic trioxide (ATO)-induced cardiac oxidative damage in mice. Thirty-six male albino mice were divided into six groups and treated intraperitoneally with normal saline (group 1), ATO (5 mg/kg; group 2), PTX (100 mg/kg; group 3), and different doses of PTX (25, 50, and 100 mg/kg; groups 4, 5, and 6, respectively) with ATO. After four weeks, the blood sample was collected for biochemical experiments. In addition, cardiac tissue was removed for assessment of oxidative stress markers and histopathological changes (such as hemorrhage, necrosis, infiltration of inflammatory cells, and myocardial degeneration). The findings showed that ATO caused a significant raise in serum biochemical markers such as lactate dehydrogenase (LDH), creatine phosphokinase (CPK) and troponin-I (cTnI), glucose, total cholesterol (TC), and triglyceride (TG) levels. In addition to histopathological changes in cardiac tissue, ATO led to the significant increase in cardiac lipid peroxidation (LPO) and nitric oxide (NO); remarkable decrease in the activity of cardiac antioxidant enzymes such as catalase (CAT), superoxide dismutase (SOD), and glutathione peroxidase (GPx); and the depletion of the total antioxidant capacity (TAC) and total thiol groups (TTGs). PTX was able to reduce the increased levels of serum cardiac markers (LDH, CPK, cTnI, TC, and TG), cardiac LPO, and improve antioxidant markers (TAC, TTGs, CAT, SOD, and GPx) alongside histopathologic changes. However, no significant changes were observed in elevated serum glucose and cardiac NO levels. In conclusion, the current study showed the potential therapeutic effect of PTX in the prevention of ATO-induced cardiotoxicity via reversing the oxidative stress.

## 1. Introduction

Arsenic is an environmental contaminant that is widely widespread in water, soil, and air due to its industrial and agricultural applications [1]. The epidemiologic evidence showed that high-chronic arsenic exposure has been associated with hepatorenal failure and cardiovascular disorders [2–4]. However, arsenic compounds have been used to treat various diseases from the past to the present [5].

Arsenic trioxide (ATO) is an effective chemotherapeutic drug used in the treatment of acute promyelocytic leukemia (APL), but its usage has been limited because of cardiovascular side effects, such as ventricular tachycardia, QT prolongation, torsade de pointes, and sudden cardiac death [6, 7]. These side effects can be caused through mitochondrial dysfunction and excess generation of reactive oxygen species (ROS) [8], functional changes of ion channels, and disrupted balance of intracellular and extracellular ions [9].

Phosphodiesterase inhibitors block one or more subtypes of the phosphodiesterase enzymes (PDEs), thereby preventing the inactivation of the cAMP and/or cGMP in various cells. In recent years, the antioxidant and anti-inflammatory properties of phosphodiesterase inhibitors have been considered in several studies [10–12]. For instance, Mohammadi et al. (2011) showed that selective phosphodiesterase inhibitors could increase survival of Langerhans islets by preventing free radical formation [13]. Moreover, sildenafil, as phosphodiesterase 5-selective inhibitor, can have beneficial role in improvement of toxicities caused via cadmium [14] and lead acetate [15].

Pentoxifylline (PTX), as a methyl xanthine derivative and nonselective PDE, is commonly used to treat intermittent claudication and peripheral vascular diseases, reducing platelet aggregation and improving red blood cell deformability [16]. Recent evidence showed that PTX inhibits ROS generation and improves capillary circulation and tissue oxygenation in various organs. For instance, Yao et al. (2016) showed that PTX could prevent intermittent hypobaric hypoxia induced-oxidative stress in testicular tissue by maintaining redox homeostasis [17]. Zhang et al. (2005) reported that PTX might be beneficial in reducing hydrogen peroxide induced embryo injury and improve in vitro fertilization (IVF) outcome [18]. Additionally, the findings of Egin et al. (2016) indicate the effective effects of PTX on oxidative stress reduction in the abdominal compartment syndrome animal model [19].

Despite the antioxidant properties of PTX, there is no evidence of the therapeutic potential of this drug on ATO-induced cardiotoxicity. Therefore, the current study was designed to assess the PTX effects on the oxidative damage induced by ATO in the heart tissue of mice.

## 2. Materials and Methods

Pentoxifylline, 2,4,6-tripyridyl-s-triazine (TPTZ), 1,1,3,3-tetramethoxypropane, bovine serum albumin (BSA), sulfanilamide, 5,5′dithiobis-2-nitro benzoic acid (DTNB), 2-thiobarbituric acid (TBA), and N-(1-naphthyl) ethylenediamine dihydrochloride were obtained from Sigma-Aldrich Chemical Company (St. Louis, MO, USA). Arsenic trioxide powder was purchased from Merck (Darmstadt, Germany).

### 2.1. Animals and Experimental Protocol

Thirty-six male albino mice (25 ± 2.5 g) ranging from 1 to 2 months in age were obtained from the animal house of Hamadan University of Medical Sciences (HUMS). The animals were kept in standard cages at suitable temperature (23 ± 2°C), 12/12 h light/dark cycle, and relative humidity 50% and received a standard diet and water *ad libitum*. The ethical concerns of animals' experiments were considered carefully, and its protocol was approved by the HUMS ethics review board (Ethical code number: IR.UMSHA.REC.1397.463).

In this study, the toxic dose of ATO 5 mg/kg/day was used based on the animal model proposed by Li et al. (2002) [20]. In addition, based on pilot studies, the dosage range of PTX was considered 25-100 mg/kg/day.

Accordingly, the mice were divided randomly into six groups of six each and treated for four consecutive weeks by intraperitoneal (i.p.) injection as follows:

Group 1: the mice received normal saline (control group)

Group 2: the mice received ATO (5 mg/kg/day)

Group 3: the mice received PTX (100 mg/kg/day)

Group 4: the mice received ATO (5 mg/kg/day) + PTX (25 mg/kg/day)

Group 5: the mice received ATO (5 mg/kg/day) + PTX (50 mg/kg/day)

Group 6: the mice received ATO (5 mg/kg/day) + PTX (100 mg/kg/day)

It should be noted that groups 4-6 were treated with different doses of PTX 1 h before ATO administration. In addition, the highest dose of PTX (100 mg/kg) was considered to show its safety in group 3. Twenty-four hours after the completion of treatment, each animal was weighed and anesthetized by ketamine (50 mg/kg) and xylazine (10 mg/kg), and its blood sample was taken through cardiac puncture. Then, blood sample was centrifuged (at 3000 g, 10 min), and its serum was kept at -20°C for the biochemical analysis. Furthermore, the heart was removed for preparation of tissue homogenate (10%, *w*/*v*). Briefly, half of the heart tissue was homogenized with phosphate-buffered saline (50 mM, pH 7.3) and centrifuged at 3000 g, 10 min at 4°C. Finally, its supernatant was removed for the biochemical experiments. Another part of tissue was fixed in 10% formaldehyde solution for histopathological analysis.

### 2.2. Determination of Glucose and Total Triglyceride and Cholesterol

Glucose, total cholesterol, and triglyceride serum levels were determined using commercial kits (Pars Azmoon, Tehran kit, Iran).

### 2.3. Lactate Dehydrogenase Assay

Lactate dehydrogenase (LDH) activity in serum sample was measured by determining the rate of oxidation of NADH by an enzymatic colorimetric kit (Pars Azmoon Co., Tehran, Iran). The absorbance change per minute was detected at 340 nm using spectrophotometric instrument (Analytik Jena Specord 50 Plus), and its results were expressed as U/L.

### 2.4. Creatine Phosphokinase Assay

The activity of serum creatine phosphokinase (CPK) was assayed by an enzymatic colorimetric kit (Pars Azmoon Co., Tehran, Iran). Based on the kit's procedure, creatine kinase converts creatine into ADP and phosphocreatine. The absorbance change per minute was detected at 340 nm, and its data were expressed as U/L.

### 2.5. Troponin-I Assay

Cardiac troponin-I (cTnI) levels in serum samples were assayed by Enzyme Linked-Immuno-Sorbent Assay (ELISA) kit, according to the manufacturer's instructions (Shanghai Crystal Day Biotech Co., LTD, China).

### 2.6. Lipid Peroxidation Assay

Cardiac lipid peroxidation was measured via the reaction of TBA with active-aldehyde intermediates such as MDA. Briefly, heart homogenate supernatant (100 *μ*l) was mixed with 500 *μ*l reagent containing TBA (0.2%) in H_2_SO_4_ (0.05 M) and subsequently heated for 30 min at 100°C in boiling water bath [21, 22]. The peak absorbance was detected at 532 nm against different concentration of MDA as the standard, and its results reported as nmol/mg protein.

### 2.7. Total Antioxidant Capacity Assay

The total antioxidant capacity (TAC) was determined in the heart homogenate supernatant by measuring the reduction of Fe^3+^-TPTZ complex to the Fe^2+^-TPTZ by a reductant at low pH [22, 23]. Briefly, a reagent was prepared by mixing 20 mM FeCl_3_, acetate buffer (300 mM, pH 3.6), and TPTZ (10 mM) in 40 mM HCL, in the ratio 1 : 10 : 1. In the next stage, 20 *μ*l of sample and 200 *μ*l reagent were mixed and incubated for 15 min. The maximum absorbance of Fe^2+^-TPTZ complex was detected at 593 nm against standard curve. Results were reported as nmol/mg protein.

### 2.8. Determination of Total Thiol Group (TTGs)

Total thiol groups (TTGs) were assayed in heart homogenate supernatant using DTNB reagent [24]. Briefly, 200 *μ*l of Tris-EDTA buffer solution (0.25 M Tris base, 20 mM EDTA, pH 8.2) and 10 *μ*l of sample were mixed together in microplate well, and its initial absorbance was detected at 412 nm. Then, 10 *μ*l of DTNB reagent (10 mmol/l in methanol) was added and incubated at 37°C for 15 min. The final absorbance of each samples (A2) and also DTNB blank (B) was detected again at 412 nm. The thiol contents were calculated by reduced glutathione as standard and presented as nmol/mg protein.

### 2.9. Nitric Oxide Assay

Nitric oxide (NO) was determined in heart homogenate supernatant by Griess reagent (1% sulfanilamide, 0.1% NED, and 2.5% phosphoric acid) as described by Nili-Ahmadabadi et al. [21]. Briefly, 100 *μ*l of sample and 100 *μ*l reagent were mixed in microplate well and incubated for 15 min at 37°C. The optimum absorbance was detected at 520 nm against different concentration of sodium nitrate solution as the standard. The results reported as nmol/mg protein.

### 2.10. Catalase Assay

The cardiac catalase (CAT) activity was determined by detecting the rate of decomposition of hydrogen peroxide (H_2_O_2_) by a UV-Vis spectrophotometric system at 240 nm. CAT activity unit (U/mg protein) was defined as 1 *μ*mol of H_2_O_2_ disappearance/min/mg protein [25].

### 2.11. Superoxide Dismutase Assay

The cardiac superoxide dismutase (SOD) activity was determined according to the kit brochure from ZellBio GmbH Company, Germany. In this experiment, SOD activity unit (U/mg protein) was defined as the amount of enzyme that catalyzes decomposition of 1 *μ*mole of superoxide radical anions to H_2_O_2_ and oxygen molecules in one minute.

### 2.12. Glutathione Peroxidase Assay

The cardiac glutathione peroxidase (GPx) activity was assayed according to the kit brochure from ZellBio GmbH Company, Germany. In this experimentation, GPx activity unit (U/mg protein) was defined as the amount of enzyme that catalyzes the oxidation of 1 *μ*mole NADPH per minute.

### 2.13. Protein Assay

At the end of each experiment, protein level of heart homogenate supernatant was measured by Bradford method that is based on an absorbance shift of the dye Coomassie Brilliant Blue G-250 at 595 nm.

### 2.14. Histopathological Analysis

The cardiac tissue was fixed in 10% formaldehyde solution at least 24 h before histopathological examination. The paraffin-embedded block was prepared using automatic tissue processor, and then, samples cut into 4-6 *μ*m thick sections by a rotating microtome [26]. After staining cardiac tissue by hematoxylin and eosin (H&E) dye, stained samples were evaluated under light microscope (Olympus CX31 microscope). After examination under screening power (40x), we examined at least 20 LPF (low power field, 100x) of each slide searching for any area of necrosis, hemorrhage, inflammation, and myocardial degeneration. Percent of abnormal findings in each LPF was roughly estimated with eye examination and the final number considered by taking average of results of different fields. The abnormal results were confirmed by HPF (high power field, 400x) examination just in case. It should be noted that the microscopic observations were scored as 0 (0%), 1 (1–25%), 2 (26–50%), 3 (51–75%), and 4 (76–100%) according to the percentage of histopathological changes.

### 2.15. Statistical Analysis

The data were analyzed by the GraphPad Prism software, version 6.0, and presented as mean ± standard error of the mean (SEM). The statistical differences between values were compared by one-way analysis of variance (ANOVA) followed by Tukey's post hoc test for quantitative variables. The significance degree was set at *P* < 0.05.

## 3. Results

### 3.1. Animal Body and Tissue Weight

As shown in Table 1, a significant decrease was observed in weight gain in the ATO group compared to the control group (*P* < 0.05). No significant changes were found in heart weight/body weight index in different groups.

### 3.2. Serum Levels of Glucose, Total Triglyceride, and Cholesterol

**Figure 1:**
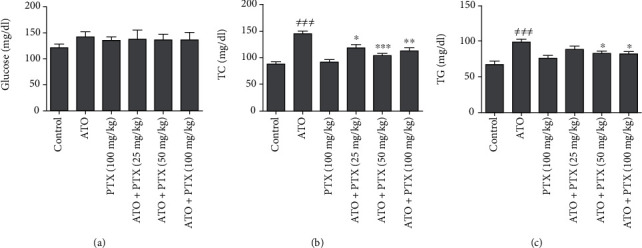
Effect of PTX on glucose and lipid serum levels in ATO-exposed mice. Statistical analysis used one-way ANOVA with Tukey's test. The results are expressed as means ± SEM, *n* = 6 for each group. ^≠≠≠^*P* < 0.001 vs. control group; ^∗^*P* < 0.05, ^∗∗^*P* < 0.01, and ^∗∗∗^*P* < 0.001 vs. ATO group. Glucose (a); TC: total cholesterol (b); TG: total triglyceride (c); ATO: arsenic trioxide (equal 5 mg/kg); PTX: pentoxifylline.

As shown in Figure 1, administration of ATO significantly raised total cholesterol and triglyceride serum levels in comparison to the control group (*P* < 0.001 and *P* < 0.001, respectively). PTX was able to reduce the increased levels of triglyceride at the employed doses of 50 and 100 mg/kg (*P* < 0.05) and total cholesterol serum levels at the doses of 25, 50, and 100 mg/kg (*P* < 0.05, *P* < 0.001, and *P* < 0.01, respectively). No significant changes were observed in the glucose serum level in the treatment groups.

### 3.3. Serum Levels of Cardiac Markers

**Figure 2:**
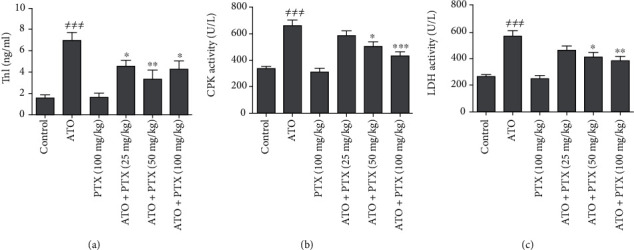
Effect of PTX on serum cardiac markers in ATO-exposed mice. Statistical analysis used one-way ANOVA with Tukey's test. The results are expressed as means ± SEM, *n* = 6 for each group. ^≠≠≠^*P* < 0.001 vs. control group; ^∗^*P* < 0.05, ^∗∗^*P* < 0.01, and ^∗∗∗^*P* < 0.001 vs. ATO group. TnI: troponin-I (a); CPK: creatine phosphokinase (b); LDH: lactate dehydrogenase (c); ATO: arsenic trioxide (equal 5 mg/kg); PTX: pentoxifylline.

As shown in Figure 2, the administration of ATO could remarkably increase cTnI (*P* < 0.001), CPK (*P* < 0.001), and LDH (*P* < 0.001) serum levels in comparison with the control group. PTX administration could decrease the serum levels of LDH and CPK, at the doses of 50 and 100 mg/kg, in mice exposed to ATO. In addition, a significant decrease was found in cTnI levels following treatment with all doses of PTX compared to ATO group.

### 3.4. Cardiac Oxidative Stress Biomarkers

**Figure 3:**
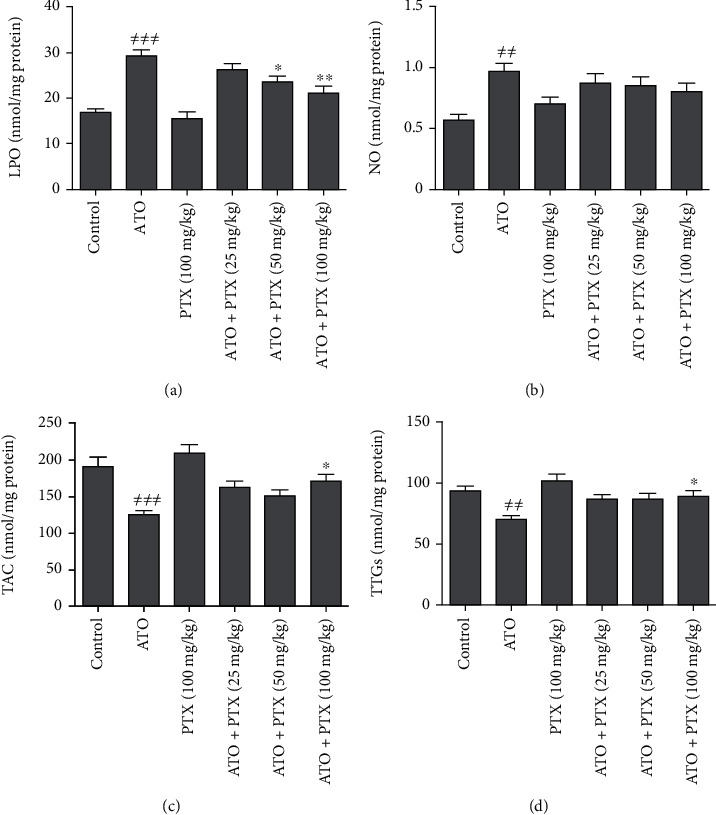
Effect of PTX on cardiac oxidative stress markers in ATO-exposed mice. Statistical analysis used one-way ANOVA with Tukey's test. The results are expressed as means ± SEM, *n* = 6 for each group. ^≠≠^*P* < 0.01 and ^≠≠≠^*P* < 0.001 vs. control group; ^∗^*P* < 0.05 and ^∗∗^*P* < 0.01 vs. ATO group. LPO: lipid peroxidation (a); NO: nitric oxide (b); TAC: total antioxidant capacity (c); TTGs: total thiol groups (d); ATO: arsenic trioxide (equal 5 mg/kg); PTX: pentoxifylline.

Following ATO administration, the levels of LPO (*P* < 0.001) and NO (*P* < 0.01) were increased, and TAC (*P* < 0.001) as well as TTG (*P* < 0.01) levels were decreased in heart tissues compared to the control group. PTX at dose 100 mg/kg significantly improved TTGs and TAC of heart tissue compared to the ATO group (*P* < 0.05). In addition, PTX could decrease cardiac lipid peroxidation at doses 50 and 100 mg/kg (*P* < 0.05 and *P* < 0.01, respectively). No significant changes were observed in the cardiac NO level in the treatment groups compared to the ATO group (Figure 3).

### 3.5. Cardiac Antioxidant Enzymes

**Figure 4:**
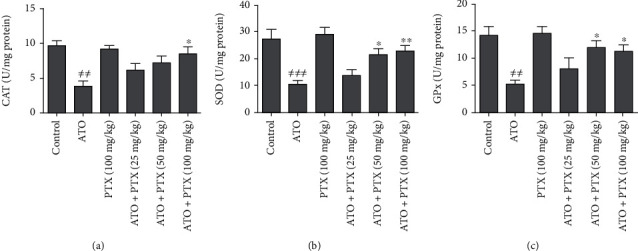
Effect of PTX on cardiac antioxidant enzymes in ATO-exposed mice. Statistical analysis used one-way ANOVA with Tukey's test. The results are expressed as means ± SEM, *n* = 6 for each group. ^≠≠^*P* < 0.01 and ^≠≠≠^*P* < 0.001 vs. control group; ^∗^*P* < 0.05 and ^∗∗^*P* < 0.01 vs. ATO group. CAT: catalase (a); SOD: superoxide dismutase (b); GPx: glutathione peroxidase (c); ATO: arsenic trioxide (equal 5 mg/kg); PTX: pentoxifylline.

As shown in Figure 4, the administration of ATO significantly decreased cardiac antioxidant enzymes activity including CAT (*P* < 0.01), SOD (*P* < 0.001), and GPx (*P* < 0.01) in comparison with the control group. PTX at dose 100 mg/kg significantly increased CAT activity of heart tissue compared to the ATO group (*P* < 0.05). In addition, PTX could improve cardiac SOD and GPx activity at doses 50 and 100 mg/kg.

### 3.6. Histopathological Changes

**Figure 5:**
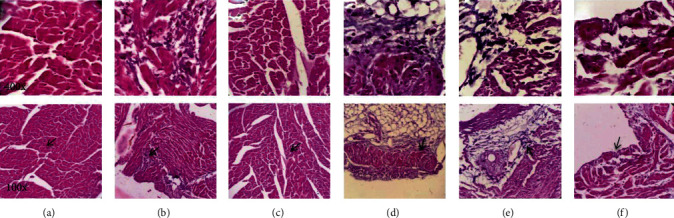
Photomicrographs of cardiac tissue in different groups: (a) control group; (b) ATO; (c) PTX (100 mg/kg); (d) ATO + PTX (25 mg/kg); (e) ATO + PTX (50 mg/kg); (f) ATO + PTX (100 mg/kg). The samples were dyed by hematoxylin and eosin. Original magnification of upper row photomicrographs is 400x and lower row photomicrographs 100x. Cardiac tissue samples of the control and PTX groups (a, c) did not show pathologic alterations, and normal myocytes with clear nuclei were observed. Coagulative necrosis and inflammation were detected in the cardiac samples of the ATO-exposed mice (b). In the treatment groups (d–f), some pathologic alterations, such as coagulative necrosis and inflammation, were decreased in a dose-dependent manner. ATO: arsenic trioxide (equal 5 mg/kg); PTX: pentoxifylline.

As summarized in Table 2, coagulative necrosis, infiltration of inflammatory cells, focal hemorrhage, and myocardial degeneration were observed in cardiac tissue of ATO-treated mice. PTX reduced some pathologic changes, such as necrosis and inflammation, in a dose-dependent manner (Figure 5).

## 4. Discussion

The present study suggests more evidence to support the involvement of oxidative stress in the pathogenesis of ATO-induced cardiotoxicity. Additionally, the results revealed the link between the antioxidant effects of PTX and its therapeutic potential against cardiac oxidative damage induced by the ATO.

Dyslipidemia is one of the most important risk factors in cardiovascular disease that can be characterized by increased triglyceride and/or cholesterol [27]. In this study, ATO-induced hypercholesterolemia may be due to increased *β*-hydroxy *β*-methylglutaryl-CoA (HMG-CoA) reductase activity, as reported by Afolabi et al. (2015) [28]. In addition, arsenic can inhibit the elimination of cholesterol from the body by inhibiting enzyme of cholesterol 7*α*-hydroxylase and preventing the biosynthesis of bile acids [28, 29]. There is little evidence regarding the influence of PTX on lipid profile. Previously, Tani et al. have shown that cilostazol, a selective type 3 phosphodiesterase inhibitor, may decrease serum triglycerides and increase HDL cholesterol in diabetic rats by increasing LPL activity. Their findings suggested that raised cAMP stimulates hydrolyzes triglycerides in lipoproteins by the release of lipoprotein lipase (LPL) from adipocytes, which may explain the reduction of serum triglyceride levels [30].

It is documented that increased levels of LDH, CPK, and cTnI in blood serum are considered as reliable diagnostic markers of myocardial toxicity [5, 31]. cTnI is cardiac regulatory protein that controls the calcium-mediated interaction between myosin and actin [32]. This protein is known as the specific and sensitive marker for the diagnosis of myocardial dysfunction [31]. LDH is a cytosolic enzyme, which is existent in various tissues involved in glycolytic pathway [33].

In the current findings, ATO intoxication caused a significant increase in the cTnI, LDH, and CPK serum levels that might due to changes in the plasma membrane integrity of cardiac myocytes and subsequently their leakage into the blood serum [34, 35]. In addition, the previous studies showed that the release of cTnI from myocardial tissue was proportional to the size and extent of tissue damage and systolic dysfunction [36, 37]. Administration of PTX significantly decreased the cTnI, LDH, and CPK serum levels as well as necrosis and inflammation in cardiac tissue towards normal in ATO-treated experimental mice. In agreement with our pathological observations, the decrease in the LDH and CPK serum levels showed a dose-dependent protection. This may be due to the membrane stabilizing effect of PTX on the myocardium, improving the cardiac damage and thereby limiting the leakage of these enzymes from the myocardial tissue. Improvements of capillary circulation and tissue oxygenation are well-known mechanisms of PTX that may be involved in preventing cardiac oxidative damage caused by ATO.

Oxidative stress (OS) is the consequence of an imbalance between antioxidant systems and reactive oxygen/nitrogen species (ROS/RNS) involved in cellular damage [38]. Manna et al. (2008) and Sun et al. (2016) studies showed that ROS/RNS are generated during inorganic arsenic metabolism in various cells [25, 39]. In this regard, our data revealed heart LPO and NO production were raised in response to ATO while cardiac TTG and TAC levels were reduced, which is in line with Hemmati et al. (2008) and Binu et al. (2017) studies [5, 40]. Overall, LPO is one of the characteristic features of OS related to arsenic toxicity, which is due to oxidative degradation of polyunsaturated acids in the cell membrane [41]. Arsenic increases the amount of free iron by releasing iron from ferritin molecule. Free iron through the Fenton reaction causes excessive production of ROS and subsequent increase in lipid peroxidation [42]. PTX was able to reduce the level of LPO in the heart tissue, which may be related to decrease the ROS generation in cardiac tissue. The part of the antioxidant effects of PTX can be attributed to its effects on reducing the activation of neutrophils, because activated neutrophils can produce superoxide radicals through NADPH oxidase [16]. In addition, PTX, an effective inhibitor of superoxide anion generation, is likely to affect the initiation and/or propagation of LPO [43]. This medicine can reduce the production of hydroxyl and superoxide radicals by inhibiting xanthine oxidase [19].

NO is an important mediator which plays a key role in the regulation of various cells. However, actions of NO are multifaceted, and its excessive production can lead to nitrosative stress [44]. Following administration of ATO, increased NO may be associated with the induction of nitric oxide synthase, which is in agreement with the findings of Kesavan et al. (2014) [45]. The reaction of NO and superoxide anion creates peroxynitrite radicals. These radicals aggravate the cellular damage through lipid peroxidation, necrosis, and apoptosis by nitration of tyrosine residues on tissue proteins [46]. There is different evidence regarding the effects of PTX on NO production. Some of these studies have suggested the inducible effects of PTX, and some have indicated its inhibitory effects on NO production. For instance, Beshay et al. showed that PTX suppress nitric oxide synthase in macrophages and its changes correlated with cellular cAMP levels [47]. In this study, PTX did not show any inhibitory effects on ATO-induced nitrosative stress when the cardiac NO levels were evaluated.

Thiol-based antioxidant system plays the main role of cellular defense against ROS/RNS-mediated oxidative injury [25, 38]. Thiol groups, as a catalyst in disulfide exchange reaction, scavenge the free radicals and detoxifying different xenobiotics and subsequently convert to oxidized form [25]. Our findings showed depletion of thiol-based antioxidant system in myocardium due to ATO toxicity, which is consistent with other reports [48, 49]. Previously, it has been described that ATO can be bound to the thiol groups and attenuates the cell antioxidant defense [50, 51]. In addition, there has been evidence of the arsenic destructive effects on enzymes affecting the level of the thiol groups, such as glutathione reductase and glutathione-S-transferase [25, 52].

Antioxidant enzymes, such as SOD, CAT, and GPx, are considered to be the first line of cellular defense against the destructive effects of free radicals [53]. Among these, the enzyme of SOD catalytically converts the superoxide radical anions into hydrogen peroxide (H_2_O_2_) and oxygen molecules while CAT catalyzes the decomposition of H_2_O_2_ to oxygen and water molecules. GPx can also minimize the destructive effects of H_2_O_2_ by using thiol molecules such as glutathione, as a reductant [54]. As our findings shown, reduced activity of SOD, CAT, and GPx enzymes can be related to cardiac oxidative damage induced by ATO which is in line with the other reports [25, 55]. Inhibition of SOD activity in ATO-intoxicated mice might be due to the increased generation of superoxide anions [56]. In addition, NADH coenzyme is vital to activate CAT from its inactivated form; inadequate supply of this coenzyme during ATO metabolism may be due to reason for decrease of CAT activity [57].

PTX noticeably increased SOD, CAT, and GPx activity, which may be associated with inhibition of superoxide anion generation and subsequently improvement of oxidant/antioxidant status in cardiac tissue of ATO-intoxicated mice.

Our findings show that PTX is able to increase the level of TTGs in cardiac tissue, which may be associated with increased production of active thiols such as glutathione. In this regard, Duranti et al. suggest that some of the phosphodiesterase inhibitors, such as tadalafil, may increase glutathione levels by increasing the activity of the enzyme glutathione peroxidase [58]. In addition, PTX-induced cAMP levels may induce glutathione-S-transferase expression and activity via the protein kinase A pathway, which may regulate detoxification of arsenic [59].

In conclusion, our findings indicated that PTX, especially at the dose of 100 mg/kg, was effective in improving ATO-induced dyslipidemia and cardiotoxicity. PTX could increase endogenous antioxidant defense, especially thiol-based antioxidant system, against oxidative destruction to protect heart tissue. In addition, improving oxidative/antioxidant balance in heart tissue following PTX administration could be an important cause of reducing ATO-induced pathogenic changes such as coagulative necrosis and inflammation. Therefore, this drug might be a suitable candidate to prevent cardiac complications caused by ATO in APL patients. However, these evidences need further studies.

## Figures and Tables

**Table 1:** Body and heart weight changes in studied groups.

Groups	Initial body weight (g)	Final body weight (g)	Weight gain (g)	Heart weight (g)	Heart weight/final body weight *x* 100
Control	25.7 ± 1.6	38.1 ± 1.7	12.4 ± 1.2	0.17 ± 0.01	0.45 ± 0.02
ATO (5 mg/kg)	26.1 ± 1.4	31.6 ± 2.1	5.5 ± 1.8^#^	0.15 ± 0.02	0.47 ± 0.05
PTX (100 mg/kg)	24.8 ± 2.1	35.6 ± 2.5	10.8 ± 1.4	0.17 ± 0.01	0.46 ± 0.03
ATO + PTX (25 mg/kg)	26.7 ± 1.6	32.5 ± 1.9	5.8 ± 1.2	0.15 ± 0.02	0.47 ± 0.07
ATO + PTX (50 mg/kg)	24.6 ± 1.3	30.9 ± 1.9	6.3 ± 1.6	0.15 ± 0.01	0.51 ± 0.05
ATO + PTX (100 mg/kg)	27.1 ± 1.7	33.6 ± 2.1	6.5 ± 2.2	0.16 ± 0.01	0.47 ± 0.05

The results are expressed as means ± SEM, *n* = 6 for each group. ^≠^*P* < 0.05 vs. control group. ATO: arsenic trioxide (equal 5 mg/kg); PTX: pentoxifylline.

**Table 2:** Histopathological alterations of cardiac tissue in experimental groups.

Groups	Coagulative necrosis	Infiltration of inflammatory cells	Focal hemorrhage	Myocardial degeneration
Control	0 ± 0	0 ± 0	0 ± 0	0 ± 0
ATO (5 mg/kg)	1.78 ± 0.21^###^	2.96 ± 0.33^###^	2.53 ± 0.27^###^	2.21 ± 0.14^###^
PTX (100 mg/kg)	0 ± 0	0 ± 0	0 ± 0	0 ± 0
ATO + PTX (25 mg/kg)	1.63 ± 0.18	2.91 ± 0.46	2.1 ± 0.37	1.70 ± 0.36
ATO + PTX (50 mg/kg)	1.01 ± 0.14^∗^	1.94 ± 0.29	1.20 ± 0.42^∗^	1.29 ± 0.40
ATO + PTX (100 mg/kg)	0.81 ± 0.31^∗∗^	1.42 ± 0.18^∗∗^	1.41 ± 0.12^∗^	1.47 ± 0.23

The results are expressed as means ± SEM, *n* = 6 for each group. ^≠≠≠^*P* < 0.001 vs. control group; ^∗^*P* < 0.05 and ^∗∗^*P* < 0.01 vs. ATO group. Statistical analysis used one-way ANOVA with Tukey's test. The microscopic observations were scored as 0 (0%), 1 (1–25%), 2 (26–50%), 3 (51–75%), and 4 (76–100%) according to the percentage of histopathological changes. ATO: arsenic trioxide (equal 5 mg/kg); PTX: pentoxifylline.

## Data Availability

The authors confirm that the data supporting the findings of this study are available within the article.
